# A Case Study of Applying Generative Design to Gear Wheels

**DOI:** 10.3390/ma19030565

**Published:** 2026-02-01

**Authors:** Matúš Virostko, Silvia Maláková, Melichar Kopas, Martin Mantič, Jozef Kuľka, Tibor Krenicky, František Lopot, Karel Petr

**Affiliations:** 1 Department of Engineering for Design of Machines and Transport Equipment, Faculty of Mechanical Engineering, Technical University of Kosice, Letna No. 9, 042 00 Kosice, Slovakia; matus.virostko@tuke.sk (M.V.); silvia.malakova@tuke.sk (S.M.); melichar.kopas@tuke.sk (M.K.); martin.mantic@tuke.sk (M.M.); jozef.kulka@tuke.sk (J.K.); 2 Department of Manufacturing Process Control, Faculty of Manufacturing Technologies with a Seat in Prešov, Technical University of Košice, Bayerova 1, 080 01 Prešov, Slovakia; 3 Department of Designing and Machine Components, Faculty of Mechanical Engineering, Czech Technical University in Prague, Technická 4, Prague 6, 160 00 Dejvice, Czech Republic; frantisek.lopot@fs.cvut.cz (F.L.); karel.petr@fs.cvut.cz (K.P.)

**Keywords:** generative design, spur gear, production technologies, material selection, manufacturing/technological constraints

## Abstract

This paper presents a numerical case study on the application of generative design to the shape optimisation of a spur gear body with consideration of manufacturing constraints and material properties. The presented results are obtained using numerical simulation and finite element analysis. A finite element-based generative design workflow was employed to evaluate weight reduction and stiffness performance under different manufacturing routes, including additive manufacturing, machining, and casting. The results show that the application of generative design enabled a gear-body mass reduction of up to 37.46–45.68% compared to the reference geometry while maintaining acceptable deformation levels. Designs constrained for additive manufacturing achieved the highest weight savings, whereas machining-constrained variants exhibited lower deformation values, indicating higher structural stiffness. Casting-oriented constraints resulted in more conservative geometries with locally reinforced regions. The study confirms that manufacturing constraints significantly influence the generated geometry and mechanical response, demonstrating that the manufacturing route acts as an independent design variable within the generative design process. The presented methodology provides practical guidance for the early-stage numerical optimisation of gear bodies and supports informed decision-making with respect to manufacturing technology selection.

## 1. Introduction

The continuous advancement of digital design and manufacturing technologies is fundamentally transforming the paradigms of modern mechanical engineering. Traditional computer-aided design (CAD) systems, which have been developed for decades with respect to conventional manufacturing processes, are now reaching their limits due to the rapid evolution of additive manufacturing (AM) capabilities [1]. In response, new computational methodologies such as topology optimisation (TO) and generative design (GD) have emerged, providing engineers with unprecedented flexibility in creating complex geometries that meet requirements for mechanical performance, manufacturability, and efficient material utilisation. In contrast to the traditional top-down approach driven by the designer, GD introduces a simulation-based, iterative methodology in which algorithms explore the design space within defined boundary conditions and propose optimal configurations [2].

Generative design and topology optimisation tools are increasingly being adopted in industrial applications as integral components of simulation-driven product development processes. Their integration with additive manufacturing enables the creation of lightweight structures with improved stiffness-to-weight ratios and reduced material consumption while maintaining structural integrity [3,4]. Studies have demonstrated that GD allows the redesign of components already in the early stages of development, leading to significant reductions in both weight and production time compared to conventional optimisation strategies [1,5]. This shift toward algorithmic and AI-assisted design highlights the evolving role of the engineer—from a manual shape creator to a definer of objectives, constraints, and performance criteria within computational frameworks [2,6].

Recent research demonstrates that the integration of GD and TO can yield highly efficient structural solutions across various engineering domains—from aerospace brackets [7] and robotic components [4], to wind turbine generators [3] and automotive systems [8]. Machine learning and deep learning methods further extend the capabilities of GD by enabling the development of design frameworks that learn from data and generate manufacturable solutions optimised for specific objectives such as stress, weight, and stiffness [9,10]. Reinforcement learning techniques are also being applied to generative processes, allowing the exploration of diverse topological configurations while significantly reducing computational time.

The ability of generative design to integrate manufacturing constraints directly into the design process enables engineers to account for additive manufacturing limitations—such as build orientation, overhangs, and surface quality—already in the early stages of development [11]. Bio-inspired frameworks derived from nature are being explored through topology optimisation for their exceptional mechanical properties in 3D-printed components [12].

The convergence of artificial intelligence (AI), machine learning (ML), and generative algorithms has also introduced data-driven and adaptive design strategies capable of learning from previous solutions, thereby significantly accelerating the conceptual design phase and enabling the generation and evaluation of thousands of 3D designs within integrated digital environments [13].

The growing adoption of additive manufacturing technologies complements these advancements by enabling the physical realisation of geometrically complex and performance-optimised designs. AM supports rapid prototyping, small-batch production, and the fabrication of metallic and polymer components that were previously unachievable using traditional manufacturing methods. Furthermore, the development of hybrid technologies combining additive and conventional casting techniques opens new possibilities for producing optimised parts with enhanced material utilisation and mechanical performance [14]. These approaches are particularly relevant for components such as gears and transmission elements, where performance, weight, and reliability are critical [15].

In the paper [16], the authors focus on applying topology optimisation techniques to the design of gear wheels in order to improve their structural efficiency. The study investigates methods for reducing weight while maintaining strength and reliability under operational loads. Using numerical simulations and computational modelling, the paper demonstrates how optimised material distribution can enhance performance and extend gear service life. In paper [17], the authors apply generative design to optimise spur gear geometry with the primary objective of material and weight reduction under a specific optimisation scenario. In contrast, the present study extends this approach by systematically evaluating multiple manufacturing constraints and load-definition strategies within a unified generative design framework, while preserving standardised tooth geometry to ensure reliable gear engagement. In article [18], the authors adapt a force-flow-based topology optimisation method to improve generative design processes. Numerical experiments on 2D cases confirm that integrating load-path analysis with generative design produces efficient and manufacturable geometries.

In parallel with advancements in manufacturing, research on material selection plays a crucial role in the success of generative approaches. The choice of material affects manufacturability, mechanical performance, and overall cost. Studies focusing on carbon fibre-reinforced polymers, metallic alloys, and composite filaments have shown that combining material optimisation with generative design leads to significant improvements in strength, stiffness, and sustainability, while substantially reducing material consumption and production time. In the context of gear design, understanding the interaction between tool material, cutting parameters, and heat treatment remains essential for achieving long tool life and consistent gear quality [19]. Integrating this manufacturing knowledge into generative frameworks enables the simultaneous optimisation of gear geometry, material properties, and process parameters.

Overall, the available literature highlights a clear shift from designer- and experience-driven processes toward automated, data-informed, and optimisation-oriented design methodologies. Generative design, in synergy with additive manufacturing and intelligent material selection, provides a foundation for the creation of lightweight, efficient, and manufacturable components. Despite its growing adoption, gaps remain in adapting GD methodologies for highly stressed mechanical components, such as gears, where precise engagement geometry, surface integrity, and material properties are critical for operational reliability.

This study does not aim to introduce a new generative design algorithm. Instead, it presents a systematic numerical case study focused on the application of manufacturing-aware generative design to spur gear bodies. The contribution of the work lies in the comparative evaluation of different manufacturing technologies implemented as design constraints, together with the assessment of multiple load-definition strategies within a unified generative design framework, with the objective of supporting weight-efficient and manufacturable gear design.

## 2. Materials and Methods

The main objective of this paper is to demonstrate the application of generative design in the development of a gear wheel, considering shape optimisation, selection of an appropriate material, and the choice of a suitable manufacturing technology. The research procedure was designed as a multi-stage process consisting of the following steps:

- Definition of input parameters and functional requirements;
- Application of generative design in development of the spur gear;
- Analysis and simulation;
- Comparison and selection of optimal solution.

### 2.1. Input Parameters and Geometric Characteristics of Gear Wheel

The first step in the design process of a spur gear was selection of geometric parameters that enable its manufacture using various production technologies—such as machining, forging, casting, or additive manufacturing, specifically 3D printing.

Within this selection, it was necessary to consider not only the technological limitations of the individual manufacturing methods but also the mechanical requirements of the component itself. A key criterion was the aim of maximising material savings, primarily for reasons of production efficiency, weight constraints, and environmental aspects. However, at the same time, the required stiffness of the gear wheel had to be maintained, as it directly affects the operational reliability and service life of the machine part.

The geometry of the gear wheel—including such parameters as the module, number of teeth, width of toothed rim, profile correction, and hub shape—was therefore designed to achieve a compromise between manufacturability and the functional requirements.

For the purposes of this study, the presented analysis was carried out for a spur gear with the number of teeth *z* = 71, standardised module *m* = 2.5 mm, pressure angle α = 20°, and tooth width *b* = 50 mm (if the size of connection hole is *d*_0_ = 55 mm).

Since suitability of the proposed gear body shape is evaluated according to the tooth stiffness, which is assessed through deformation analysis using the Finite Element Method (FEM), the value of load acting on the tooth side wall was set to a unit value of *F* = 1000 N.

### 2.2. Application of Generative Design in Proposal of Spur Gear

This section describes the application of generative design for the shape optimisation of a spur gear body, with the primary objective of reducing component weight while preserving the required stiffness and manufacturability. The generative design approach integrates functional requirements, mechanical loading, material properties, and manufacturing constraints within a unified numerical framework, enabling a systematic exploration of feasible design solutions.

Several commercial- and research-oriented platforms currently support generative or topology-based design workflows, including Siemens NX, PTC Creo, SolidWorks, as well as specialised topology optimisation tools. However, the present work is formulated as a case study and therefore focuses on the application of generative design implemented in Autodesk Fusion 360 (https://www.autodesk.com/products/fusion-360, accessed on 6 November 2025) as a representative computational environment capable of integrating loading conditions, manufacturing constraints, and possessing material properties. The emphasis of the study is placed on the methodological aspects of generative shape optimisation rather than on software-specific features.

The overall workflow of the generative design process applied to the spur gear consists of the following key steps:

- Definition of preserved and obstacle geometry;
- Specification of the design space;
- Application of boundary conditions and loading;
- Definition of optimisation objectives and manufacturing constraints;
- Selection of material properties as input parameters.

#### 2.2.1. Definition of Geometry, Design Space, Constraints, and Loading

The generative model of the spur gear was created based on an initial input geometry that complies with standardised geometric and functional requirements for gear design. Regions that must remain unchanged during optimisation—such as the hub area around the mounting bore and the rim beneath the gear teeth—were defined as preserved geometry to ensure structural integrity and functional compatibility. In accordance with standard design practice, the rim thickness beneath the teeth was set to at least 3.5 times the module, and minimum hub thickness was specified to ensure sufficient load-carrying capacity.

In the present study, generative design optimisation is intentionally limited to the gear body, while the tooth geometry and engagement profile are fully preserved in accordance with standardised gear design requirements. This approach ensures that the optimisation does not affect load-carrying capacity, contact conditions, or kinematic accuracy of the gear teeth, which are critical for highly stressed gear applications. The study therefore focuses on evaluating the potential of manufacturing-aware generative design for optimising the gear body under realistic loading conditions, rather than for modifying the tooth geometry itself.

**Figure 1:**
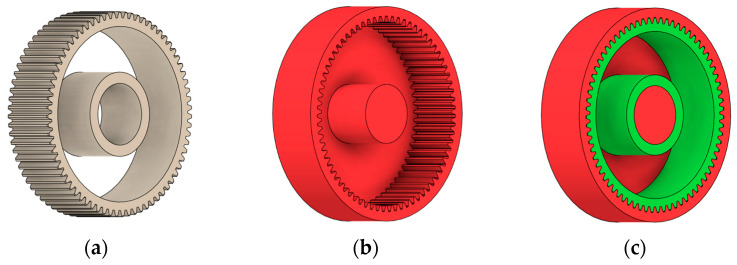
Defining of (**a**) preserved geometry of the spur gear mode; (**b**) obstacle geometry; (**c**) design space available for generative optimisation.

Obstacle geometry was introduced to restrict material removal in regions where geometric intrusion is not permissible due to assembly, functional, or technological requirements. Based on the preserved and obstacle regions, the design space was defined as the volume between the hub and the gear rim, within which material redistribution was allowed during the generative optimisation process. Figure 1 illustrates the definition of preserved geometry, obstacle regions, and the resulting design space used for generative shape exploration.

Boundary conditions were applied to simulate realistic mounting of the gear on a shaft. Selected faces of the hub bore were constrained to represent rigid fixation while allowing elastic deformation of the gear body under load.

To evaluate the influence of loading conditions on the generated geometry, three loading strategies were considered:

- Application of the maximum force to a single tooth;
- Application of the total transmitted torque distributed among the teeth;
- Conservative worst-case scenario assuming simultaneous loading of all teeth.

**Figure 2:**
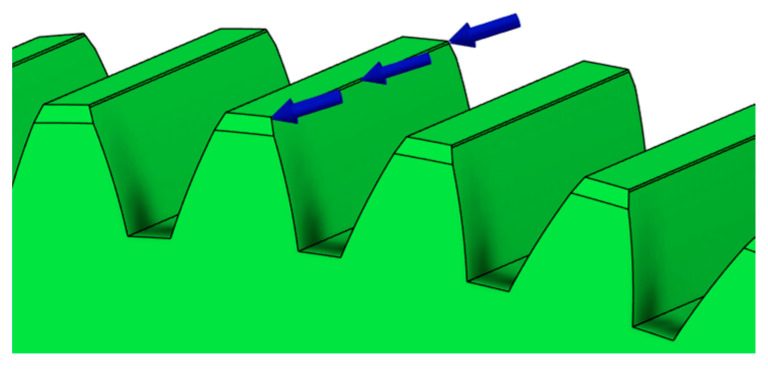
Defining the maximum force acting on single gear tooth (continuous load).

The first approach involves specifying the maximum force acting on a single gear tooth (Figure 2). The software evaluates this configuration by performing a series of individual analyses, in which each tooth of the gear wheel is considered separately. The applied force is defined as a continuous load.

**Figure 3:**
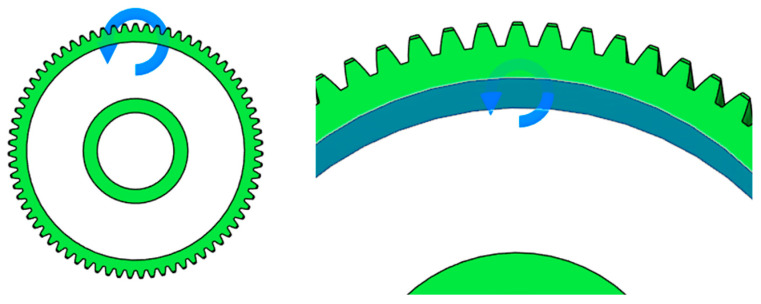
Setting of total torque acting on spur gear.

The second approach involves applying the total torque acting on the gear (Figure 3). The software then distributes this torque among the teeth according to their number and position in mesh, allowing for a more realistic and comprehensive load simulation.

**Figure 4:**
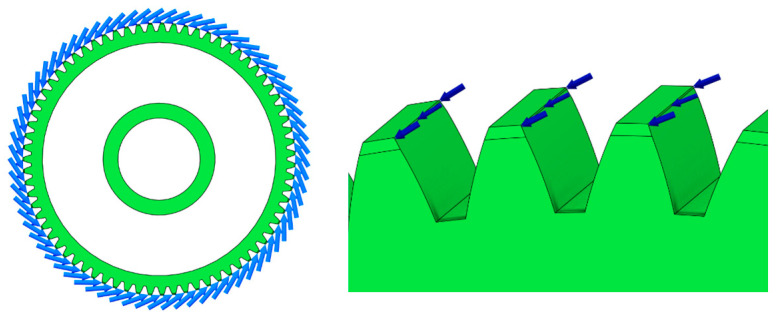
Worst-case load model—all teeth simultaneously subjected to maximum force.

The third and most conservative approach assumes that all gear teeth are simultaneously subjected to the maximum applied force (Figure 4). This loading configuration is used to evaluate the upper-bound response of the gear body and represents a worst-case scenario in terms of structural loading.

In contrast to many existing generative design studies, which typically apply a single predefined loading condition or focus primarily on shape optimisation under idealised assumptions, the present approach systematically evaluates multiple load-definition strategies within the same generative framework. By comparing single-tooth loading, torque-based loading, and a conservative worst-case scenario, the study aims to assess the sensitivity of the generated gear-body geometry to load modelling assumptions. This approach enables a more comprehensive evaluation of generative design outcomes in the context of gear-wheel applications, where load distribution and structural support conditions play a critical role.

#### 2.2.2. Optimisation Objectives and Manufacturing Constraints

The primary optimisation objective of the generative design process was the minimisation of gear-body weight while maintaining the required stiffness and strength of the gear teeth. This objective was complemented by constraints on safety factor, allowable displacement, and modal frequency to ensure functional reliability and stable operation under service loads.

Manufacturing constraints were defined to reflect different production routes applicable to gear-wheel fabrication and to ensure the practical manufacturability of the generated shapes. Within the generative design framework, three manufacturing approaches were considered: additive manufacturing, subtractive machining, and casting-based production. Each of these routes imposes specific geometric and technological limitations that directly influence the admissible design space and the resulting topology of the gear body.

In the context of this study, the analysis focuses primarily on the comparison between additive and subtractive manufacturing strategies, as these methods represent contrasting levels of geometric freedom and constraint with respect to weight reduction, stiffness, and achievable shape complexity. Casting-related constraints were considered as a reference for conventional mass-production scenarios, providing a baseline for assessing the applicability of generative design under more restrictive manufacturing conditions.

By combining mechanical performance objectives with manufacturing-specific constraints, the generative design process yields shape proposals that are not only structurally efficient but also compatible with realistic production requirements.

In this study, manufacturing technology is not considered as a post-processing option applied to an identical geometry. Instead, each manufacturing route is introduced as a set of manufacturing constraints within the generative design process, which directly influences the allowable design space. As a result, different manufacturing routes lead to different generated geometries. Therefore, manufacturing technology represents an independent variable, while the resulting geometry and its mechanical response constitute dependent variables.

#### 2.2.3. Material Selection in Generative Design

Material selection within the generative design framework was performed by choosing predefined materials from the software material database, which served exclusively as input parameters for the numerical optimisation. Numerical stress and deformation analysis is an integral part of the generative design process used in this study. Therefore, material properties must be defined as input parameters for the finite element evaluation of the generated gear designs. No material design, development, or modification was performed as part of the generative process. The selected materials were used to represent typical material classes associated with different manufacturing routes considered in this case study.

As part of the present case study, specific materials commonly employed in industrial practice were selected to reflect representative material sets for each manufacturing technology. These materials were not evaluated as optimal solutions but were chosen to enable a consistent comparative assessment of generative design outcomes under different production constraints.

For additive manufacturing scenarios, polymer- and fibre-reinforced composite materials suitable for 3D printing were considered. These included glass-fibre-reinforced polyamide (PA12 GF30), carbon-fibre-reinforced polyamides (e.g., PA-CF-based materials), and engineering polymers such as polyoxymethylene (POM). These materials are typically used for lightweight gear components and functional prototypes, where reduced mass, geometric complexity, and economic efficiency are prioritised. Metallic powders for additive manufacturing were not addressed in this case study; however, their use represents a feasible option in applications where technological requirements justify their application despite higher material costs.

For subtractive manufacturing by machining, metallic materials commonly applied in gear production were selected. These comprised medium-carbon structural steel (S45C), stainless steel (SUS420J2), alloy steel intended for case hardening (SCr415), and a high-strength aluminium alloy (EN AW-7075). These materials represent a range of mechanical properties and machinability characteristics relevant to precision gear manufacturing.

Casting-related material options were included as a reference for conventional mass-production scenarios. In this case, low-carbon and alloy steels (e.g., S45C and SCr415) as well as aluminium alloys such as EN AW-7075 were considered to reflect typical industrial casting practice.

In combination with optimisation objectives and manufacturing constraints, the selected material parameters influenced admissible stress levels, deformation limits, and the resulting geometry generated by the optimisation process. This formulation allowed the generated design variants to be evaluated under comparable conditions across different manufacturing scenarios, without introducing material-specific design modifications.

## 3. Results

The geometries of spur gear bodies can vary considerably and often include features such as webs of different shapes, lightening holes, and other structural details. These elements are designed to achieve an optimal combination of weight reduction, structural stability, and manufacturing feasibility, while maintaining the functionality and operational reliability of the gear wheel.

For clarity and practical relevance, only a selected subset of generated design variants is presented in Table 1, Table 2, Table 3 and Table 4. These variants represent manufacturable, mechanically feasible, and economically reasonable solutions filtered from a larger set of automatically generated outcomes, while non-realistic, technologically infeasible, or cost-inefficient geometries were excluded from further evaluation.

### 3.1. Generation of Gear Wheel Body Geometry with Definition of Basic Geometrical Constraints

At the beginning of the investigation into the use of Autodesk Fusion 360 for designing the body of a spur gear, freedom was given to the software itself to propose an optimal solution through the principles of generative design. The aim of this approach was to verify the capability of the programme to automatically generate shapes that take into consideration the selected manufacturing method, material, and the required functional properties of the component.

The definition of the design space in this case is illustrated in Figure 1. The uncolored part of the model represents the design space itself, within which the generative design process takes place. A selection of results—that is, the generated solutions for the individual types of manufacturing technologies under torque loading of the gear wheel (Figure 3)—is presented in Table 1.

**Table 1:** Shape generation with definition of basic geometric constraints.

Manufacturing	Type of Load—Torque		
	Variant 1	Variant 2	Variant 3
Additive	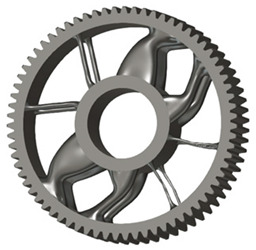	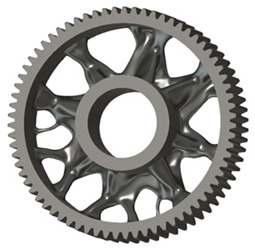	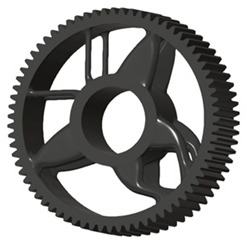
Casting	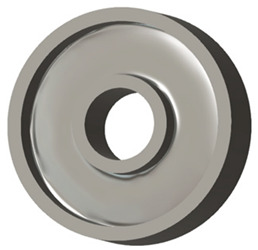	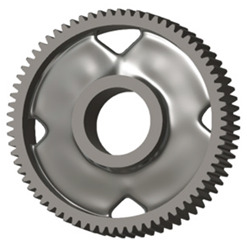	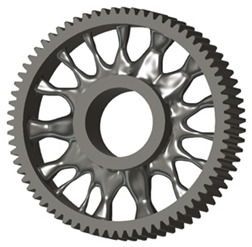
Milling 5 axis	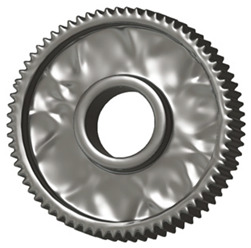	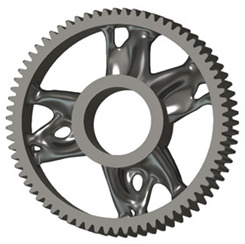	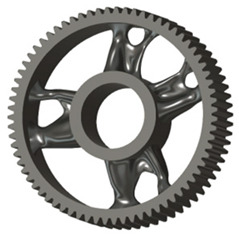

After completing the computational process, the programme generated several alternative design solutions. Table 1 presents only a selected sample of the proposed options. As shown in the presented results, the suitability of several automatically generated solutions does not always correspond to the selected manufacturing methods—certain geometries are atypical and, from a practical manufacturing standpoint, often infeasible. This illustrates that the generative programme produces many designs, among which some may be unsuitable for practical use. For this reason, the subsequent stage of the analysis involved a more precise definition of the available design space, with the aim of obtaining more realistic and technologically feasible shape proposals.

### 3.2. Design Optimisation Using Refinement of Geometric Constraints

**Figure 5:**
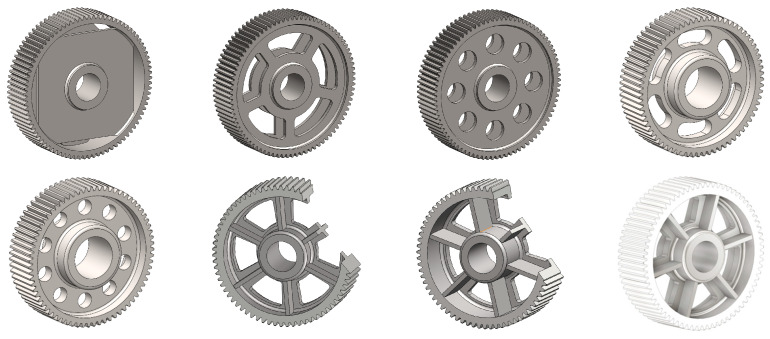
Examples of structural solutions for lightening of spur gear bodies.

In the design of a spur gear body, the primary objective is to achieve the lowest possible component weight while maintaining the required stiffness and tooth strength. In engineering practice, various structural modifications of gear bodies are therefore commonly applied to enable effective weight reduction without negatively affecting the functional performance of the gear. Among the most frequently used solutions are webs, cutouts, and lightening holes of different shapes, which ensure optimal material distribution. Typical examples of these design configurations are illustrated in Figure 5.

**Figure 6:**
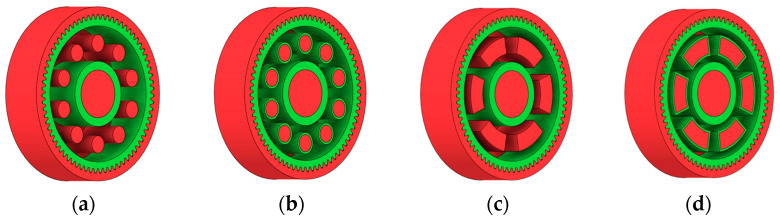
Variants of lightening a spur gear body: (**a**) ten circular holes without preserved material; (**b**) ten circular holes with material preserved around the holes; (**c**) six holes without preserved material; (**d**) six holes with material preserved around the holes.

There are several approaches to reduce the weight of a spur gear body. In this study, two specific methods were selected: weight reduction by means of ten circular holes (Figure 6a,b) and weight reduction by means of six holes (Figure 6c,d). For both types of designs, it is possible to define a material layer of a certain thickness around the holes that must be preserved. Such a constraint modifies the design space and simultaneously influences the final geometry of the gear body generated by the software. Circular holes were specifically preferred over other shapes, as their machining is more efficient and cost-effective, making this an important criterion when selecting a configuration with comparable mass reduction.

As previously mentioned, the red volumes represent regions into which the generated geometry must not extend, whereas the green volumes denote the areas of material that must be preserved. The design space corresponds to the free volume within the model where the generative algorithm is allowed to create the new shape of the gear body.

Table 2 presents selected solutions for the generated shapes of spur gear bodies with circular lightening holes. For the individual variants, different manufacturing approaches are considered, comparing options with preserved material around the lightening holes and those without such preservation. Dissymmetrical or otherwise impractical variants were excluded based on technological and functional criteria to ensure manufacturability and operational reliability.

**Table 2:** Results of generative design of the gear body with lightening holes.

Manufacturing	Lightening Options	Type of Load		
		All Teeth (Figure 4)	Torque (Figure 3)	71 Separate Studies
Additive	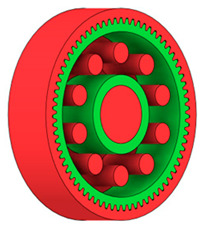	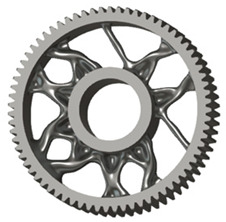	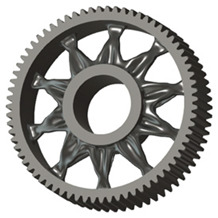	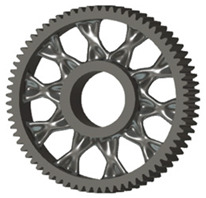
	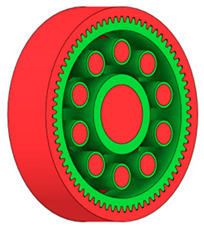	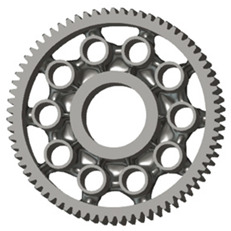	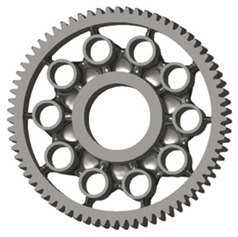	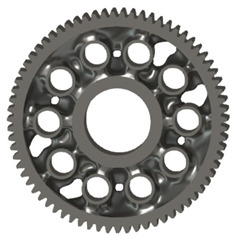
Milling 3 axis	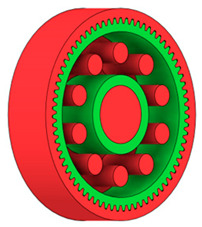	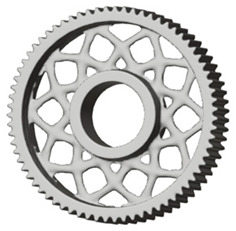	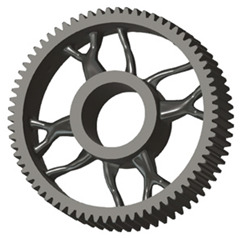	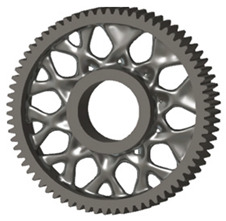
	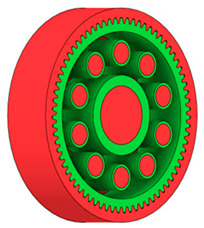	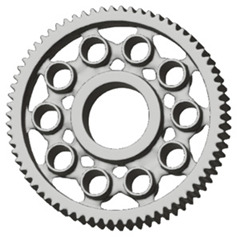	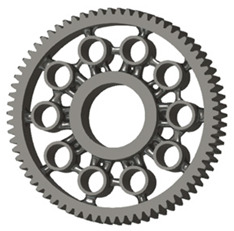	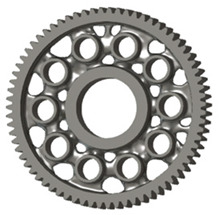
Milling 5 axis	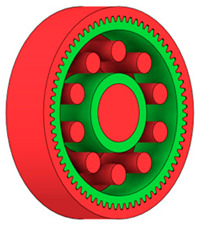	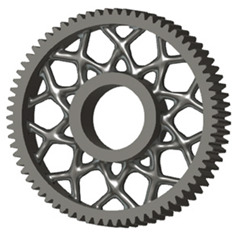	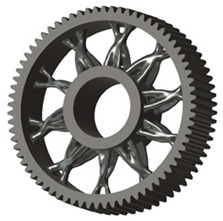	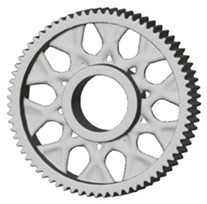
	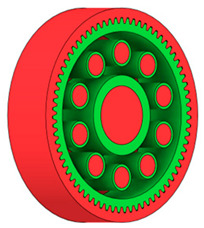	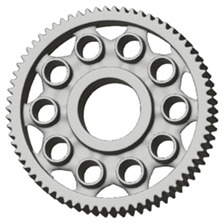	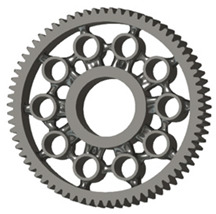	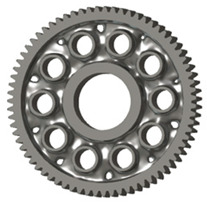
Casting	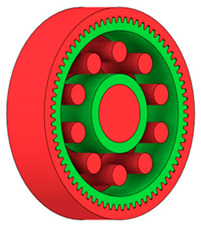	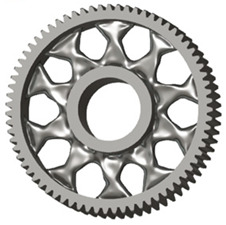	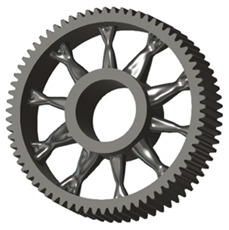	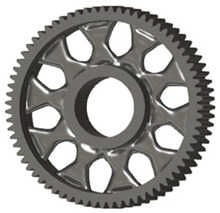
	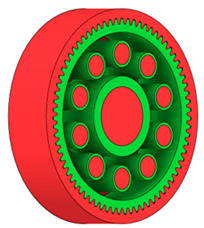	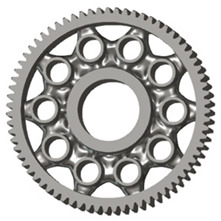	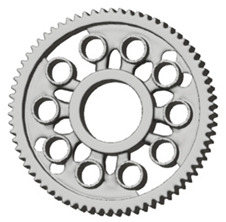	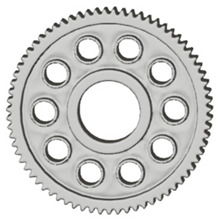

Despite the precisely defined conditions, the programme also generates solutions that are inadequate, unsuitable, or practically non-manufacturable. Some of these variants exhibit only a single axis of symmetry, while others, such as the lower-left variant in Table 3, are symmetrical but exhibit different stiffness in different directions. This suggests that the issues may not lie solely with the software or its limitations, but also with the sensitive definition of input parameters, particularly the specification of the axis of symmetry. Correctly defining the axis of symmetry and considering directional stiffness is crucial to ensure that a rotational part meets the required symmetry and is practically manufacturable. These solutions were obtained under the same input settings as the results presented in Table 2 and are summarised in Table 3.

**Table 3:** Unsuitable solutions of generated spur gear body shapes.

All Teeth (Figure 4)	Torque (Figure 3)	71 Separate Studies
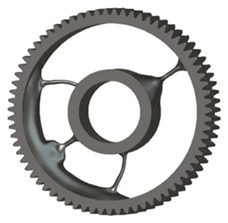	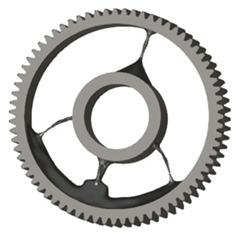	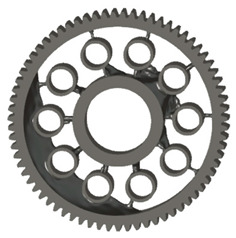
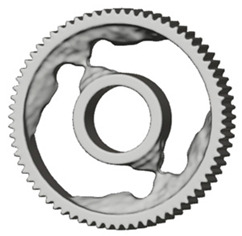	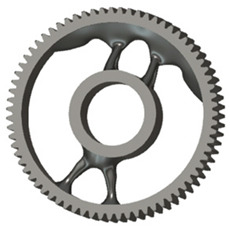	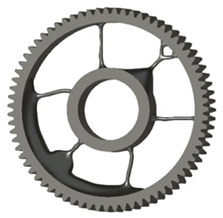

Similarly, Table 4 shows selected solutions for the generated shape of the spur gear bodies with six lightened holes (see Figure 6c,d).

**Table 4:** Results of generative design of the gear body with six lightening holes.

Manufacturing	Lightening Options	Type of Load		
		All Teeth (Figure 4)	Torque (Figure 3)	71 Separate Studies
Additive	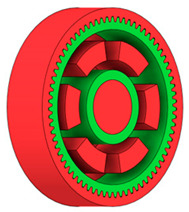	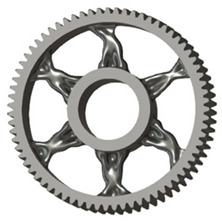	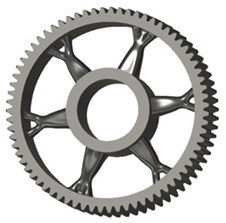	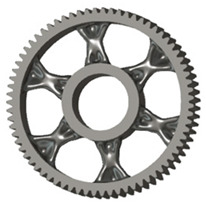
	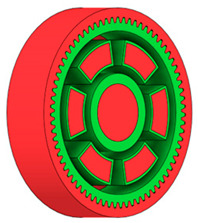	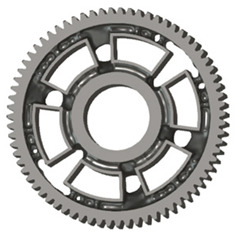	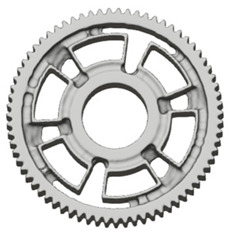	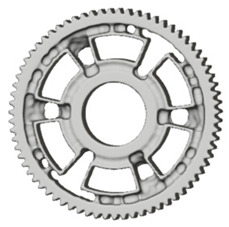
Milling 3 axis	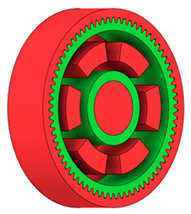	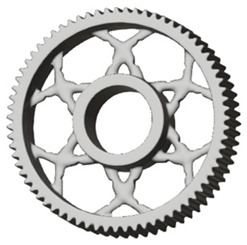	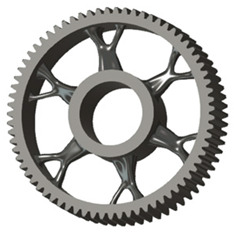	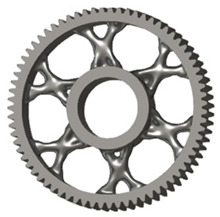
	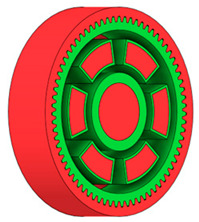	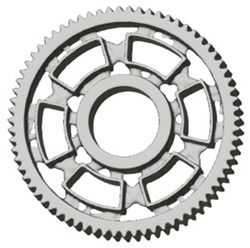	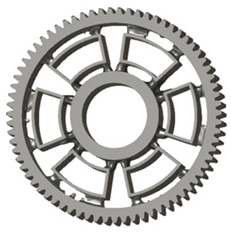	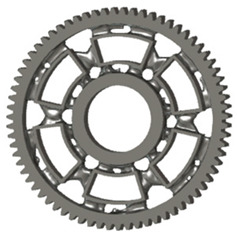
Milling 5 axis	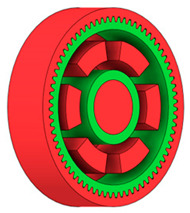	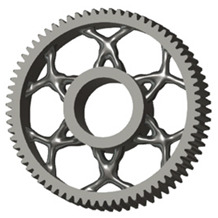	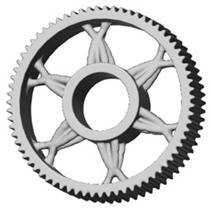	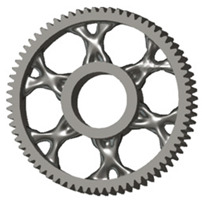
	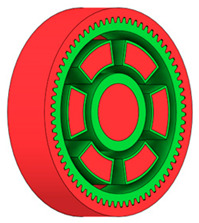	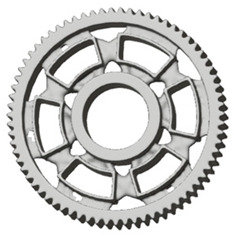	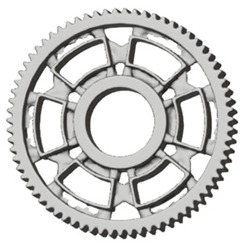	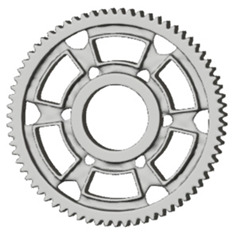
Casting	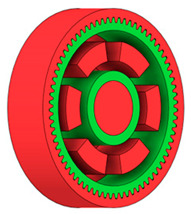	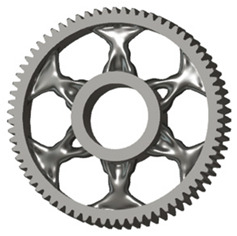	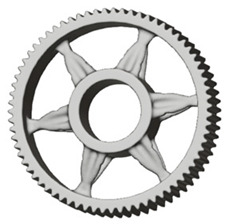	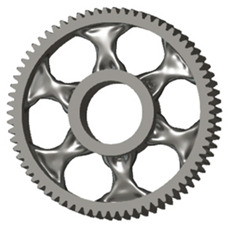
	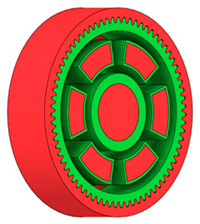	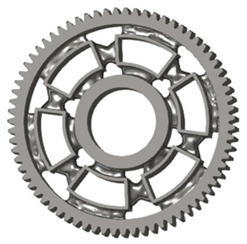	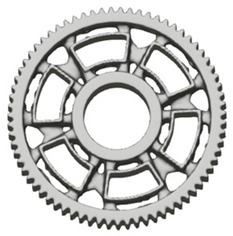	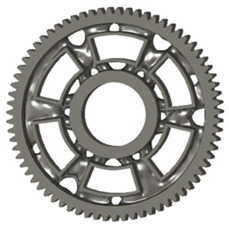

### 3.3. Design Optimisation Using Gear-Tooth Stiffness and Volume of Body Wheel Minimization

**Figure 7:**
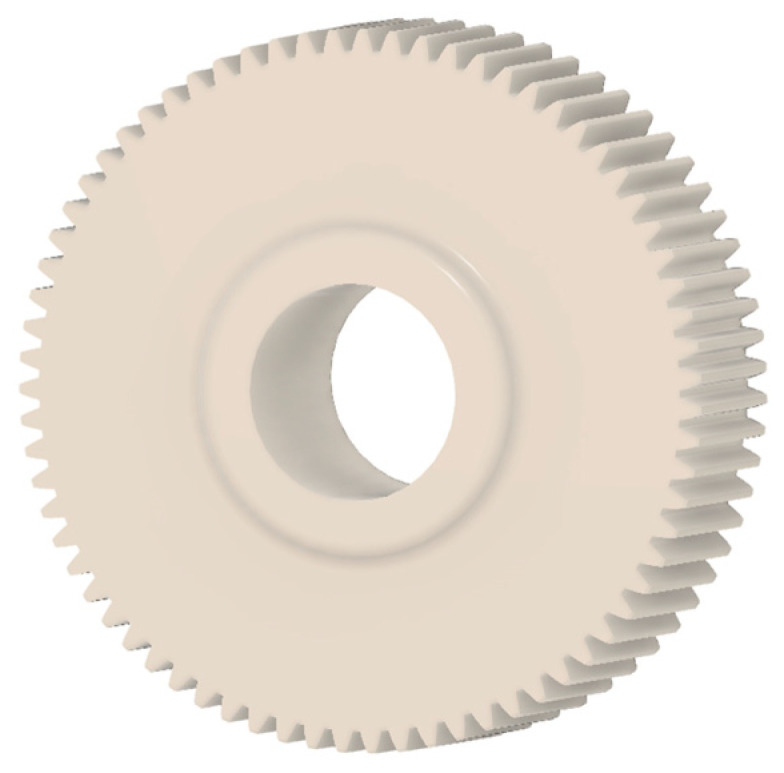
Solid gear blank before optimisation.

In this case, it was possible to monitor the change in the mass or volume of the gear wheel as the primary optimisation criterion. Since the study also considered the influence of the manufacturing method, various materials with different densities and mechanical properties were proposed, which resulted in different volumes and masses of the final designs. Therefore, the study focused on volume as the priority parameter over mass, allowing the assessment of design efficiency independently of the selected material. Based on the calculated volume of each variant, this parameter was compared with the volume of a solid (unrelieved) gear body (Figure 7), providing a unified measure of material savings. Such a comparison makes it possible to quantify the degree of structural light-weighting and to identify the most efficient geometries in terms of the volume of the wheel body.

The results indicate which regions of the gear can be lightweighted without compromising stiffness or strength, and which areas require material retention to preserve the functional characteristics. This approach provides a practical tool for decision-making regarding material selection and manufacturing technology, as a reduced volume enables a broader choice of cheaper or lighter materials. The volume value relative to the solid gear body thus becomes a key criterion in gear-shape optimisation, providing a clear metric for comparing different design variants.

During gear meshing, tooth deformations occur as a result of the transmission force acting between the tooth flanks. These deformations affect motion-transfer accuracy, transmission noise, and load distribution within the mesh. Gear mesh stiffness represents the resistance of the teeth to these deformations and defines the relationship between the applied force and the resulting deflection or displacement in the mesh.

The mesh stiffness of a gear pair is not constant—it varies throughout the meshing cycle as the contact point shifts along the tooth flanks and the number of simultaneously engaged tooth pairs changes. Determining the overall mesh stiffness is therefore a complex task that involves bending, shear, contact, and torsional deformations of the teeth, as well as the elastic effects of shafts and bearings.

To simplify the problem, the analysis often starts with evaluating the stiffness of an individual tooth subjected to a force applied at a specific point, most commonly at the tooth tip. This approach makes it possible to examine the tooth’s behaviour under a known load and to derive its bending and contact stiffness. Tooth stiffness can be characterised as the ratio of the applied force to the corresponding deformation, typically in the direction normal to the line of action.

**Figure 8:**
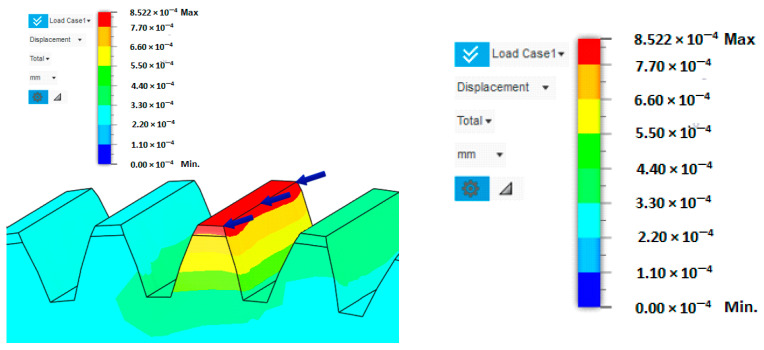
Tooth deformation solved by FEM.

In this case, tooth stiffness was determined based on the deformation induced by a load applied at the tooth tip. It was assumed that the force acts in the direction normal to the tooth flank at the contact location, which best represents the real conditions in gear meshing (Figure 8). To determine the resulting deformation, the finite element method (FEM) was employed, allowing detailed evaluation of the stress–strain state within the individual regions of the tooth. The tooth model was loaded with a unit force of *F* = 1000 N, and the stiffness was subsequently calculated as the ratio between the applied force and the corresponding deformation.

**Figure 9:**
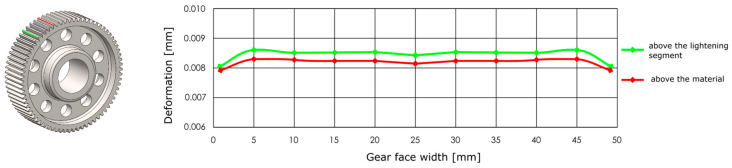
Distribution of deformations above the lightening segment and outside of it.

The deformation along the entire tooth width is neither uniform nor constant. The deflection values of the gearing vary depending on whether the loaded tooth is located directly above the lightening segment of the gear wheel body or outside its influence. When the tooth is positioned above this lightened segment, its stiffness is reduced, resulting in greater deformation compared to a tooth situated in a fully supported section of the gear wheel body. This difference is clearly visible in Figure 9, which illustrates the increased tooth deflection above the lightening segment. For this reason, the analysis was conducted considering the most unfavourable loading condition, specifically when the applied force acts on a tooth positioned above the lightening segment. This configuration represents a less stiff support for the tooth, resulting in higher deformation values and allowing the evaluation of the gear wheel under the most critical operational conditions.

**Figure 10:**
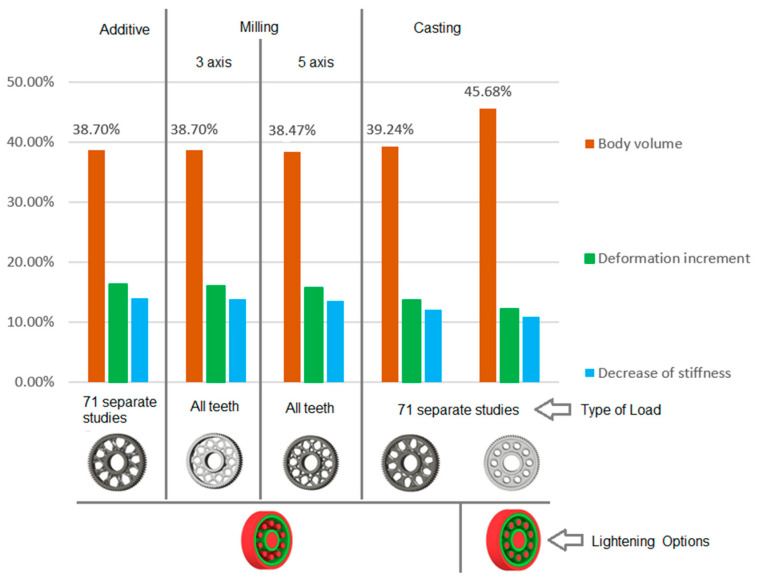
Analysis of the gear body shape with circular lightening holes depending on the manufacturing method.

Figure 10 presents the results of the analysis of the gear body shape design for models with circular lightening holes, evaluated with respect to different manufacturing methods. From the set of proposed solutions for each manufacturing technology, representative variants were selected. For these variants, the gear body volume was examined and compared to the solid (full) gear body, whose volume is considered as 100%. Additionally, the tooth deformation and stiffness above the lightening hole were analysed and compared to the deformation and stiffness of the teeth in the solid gear body. Based on this case study, it can be concluded that when using generative design for selecting the shape of a gear body, the most appropriate loading method is to apply the force simultaneously to all teeth. Alternatively, a series of individual studies for each tooth can be used. Furthermore, it is preferable to use a lightening-hole variant without preserving material around the holes.

**Figure 11:**
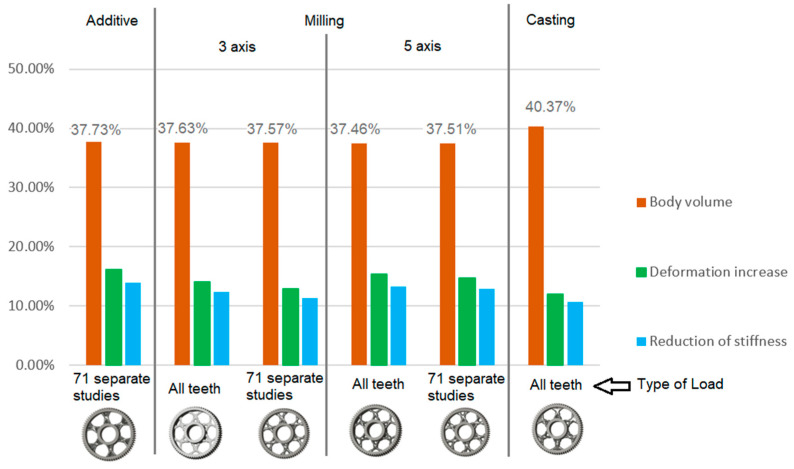
Analysis of the gear body shape with lightening holes depending on the manufacturing method.

The analysis for lightening holes of shapes other than circular is shown in Figure 11. This study also confirms the most appropriate loading method, similar to the previous case, and likewise supports the use of a lightening concept without additional material around the lightening holes. The differences in volume among the selected representative models are comparable; more significant differences occur in tooth deformation and stiffness, which depend on the influence of the body geometry and the chosen manufacturing method. It should be noted that the results for the three- and five-axis CNC machines are very similar, indicating that the more expensive five-axis machining may not provide sufficient additional benefit to justify its higher cost.

**Figure 12:**
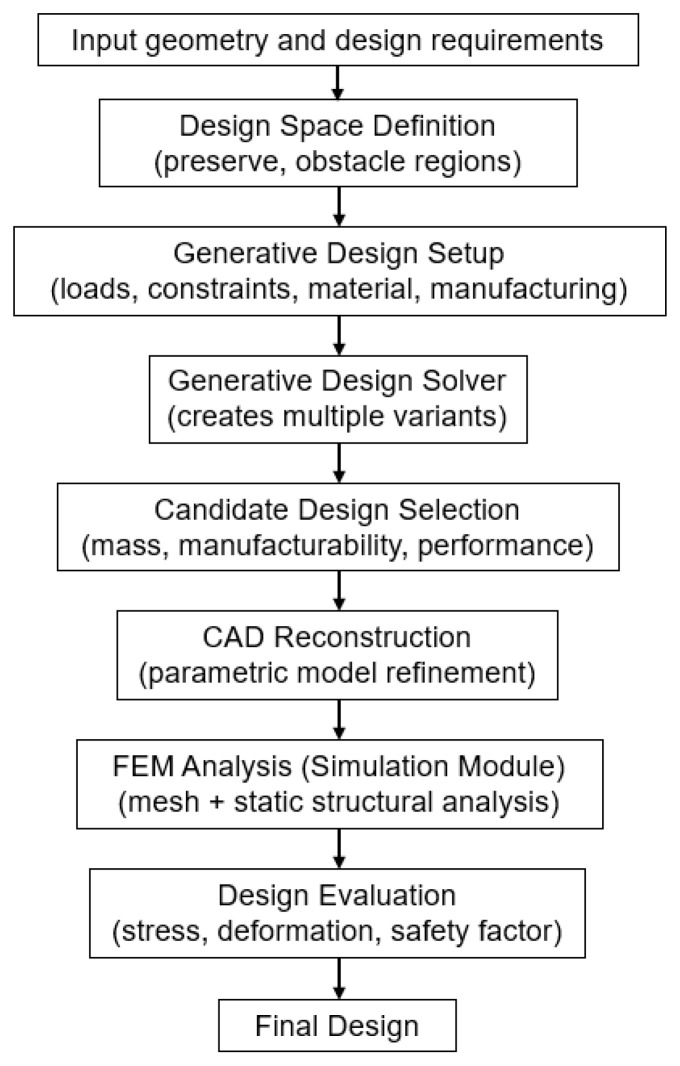
Workflow of generative design and FEM verification.

Figure 12 presents a flowchart illustrating the generative design workflow and subsequent FEM verification. The diagram includes the definition of the design space (preserve/obstacle regions), specification of boundary conditions and loads, material selection and manufacturing constraints, generation of design variants, CAD reconstruction of selected variants, and static FEM analysis. The outputs of the generative design process are converted into a parametric CAD model, which serves as the input for FEM simulation. Based on FEM results (stress, deformation, factor of safety), the variants are evaluated and compared against the design objectives.

In the design of gears, it is often desirable to minimise the volume of the gear body, resulting in material savings and a reduction in the overall weight of the transmission system. However, reduction in volume must be achieved while maintaining the required tooth stiffness and strength so that neither service life nor operational performance is compromised [20]. Geometry optimisation—such as adjusting the disc thickness, hub shape, or removing material from less-loaded regions—makes it possible to achieve an effective balance between weight reduction and mechanical resistance. Therefore, the design process relies on stiffness and stress analysis results, which identify critical areas where material can be removed without affecting functionality. Such an approach leads to a more efficient, lighter, and cost-effective gear design, suitable even for modern dynamic applications.

## 4. Discussion

The following discussion focuses on the interpretation of the numerical results obtained in this study. The emphasis is placed on the methodological application of generative design under different manufacturing and loading conditions, rather than on the development of new generative design algorithms.

The results obtained in this study demonstrate that the application of generative design to spur gear bodies leads to significantly different geometrical and mechanical outcomes depending on the imposed manufacturing constraints and loading strategies. Unlike conventional design approaches, where a single geometry is subsequently adapted to different manufacturing technologies, the generative design framework inherently couples manufacturing constraints with shape generation. As a result, each manufacturing route produces a distinct geometry optimised within its own admissible design space.

The observed differences between gear-body variants can be primarily attributed to the manufacturing constraints imposed during the generative design process. Additive manufacturing enables a high degree of geometric freedom, allowing the algorithm to redistribute material along principal load paths and form organic rib-like structures. This leads to substantial weight reduction while maintaining acceptable stiffness levels. Similar trends have been reported in generative and topology-optimised mechanical components, where additive manufacturing consistently enables more efficient material utilisation compared to conventional processes [1,3,5,7,11,21,22].

In contrast, subtractive manufacturing introduces constraints related to tool accessibility and machining directions, which limit geometric complexity. As a consequence, the generated geometries are more conservative, exhibiting thicker load-bearing regions and reduced material removal. This behaviour explains the higher stiffness and lower deformation observed for machined variants, albeit at the expense of increased mass. Comparable findings have been reported in studies focusing on topology optimisation under machining constraints, where stiffness preservation often dominates weight reduction [2,18,23,24].

Casting-related constraints were considered as a reference scenario representing conventional mass production. The results indicate that the necessity to preserve material around openings and maintain manufacturable wall thicknesses restricts the achievable level of optimisation. However, this conservative material distribution contributes to improved durability, which is consistent with industrial practice for cast gear wheels used in high-load applications [14,15,25].

From a mechanical perspective, the deformation behaviour observed in the numerical simulations reflects the interaction between load transmission paths and local material distribution. Increased deformation in regions above lightening features is expected, as these areas experience reduced cross-sectional stiffness. The generative design algorithm compensates for this effect by reinforcing load-bearing regions when permitted by manufacturing constraints [16,17,19].

The observation that material retention around lightening features limits the optimisation space without a proportional increase in stiffness may appear intuitive at first glance. However, within the generative design framework, this behaviour is not imposed explicitly by the designer but emerges from the interaction between mechanical loading, finite element-based evaluation, and manufacturing constraints. The generative algorithm systematically redistributes material to satisfy stiffness and deformation limits under the imposed load cases, revealing that further material removal in these regions leads to diminishing structural benefit. This outcome highlights the role of generative design as a quantitative tool that confirms and bounds conventional design heuristics under explicit numerical and manufacturing constraints, rather than rely on empirical intuition alone.

The comparison of different loading strategies further highlights the importance of realistic load modelling in generative design. Single-tooth loading emphasises local stiffness, while torque-based loading distributes stresses more uniformly across the gear body. The conservative worst-case loading scenario provides an upper-bound estimate of structural response, ensuring that the generated designs remain mechanically feasible under extreme conditions. Similar observations regarding the sensitivity of topology-optimised designs to load definition have been reported in the literature [4,20,26].

The purpose of material selection in this study is not to explore novel material solutions but to provide representative material classes associated with different manufacturing routes, enabling a consistent numerical comparison of generative design outcomes. Within the finite element-based generative workflow, material properties directly influence admissible stress levels and deformation limits and therefore affect the resulting geometry. By fixing material definitions within each manufacturing scenario, the analysis isolates the influence of manufacturing constraints and load-definition strategies on the generated shapes. This approach supports early-stage design decision-making by clarifying how generative design responds to combined mechanical and technological requirements, rather than by optimising material–geometry synergies for specific high-performance applications [6,19].

Although the present work is based exclusively on numerical simulations, the obtained trends are consistent with previously published studies on generative and topology-based optimisation of gear components and comparable mechanical parts. Cristian et al. [21] and Schmitt et al. [22] reported that additive manufacturing-oriented optimisation leads to higher weight reduction potential, while machining-constrained designs exhibit superior stiffness characteristics. Wang et al. [23] and Zhang et al. [24] similarly demonstrated that manufacturing-aware optimisation significantly influences both geometry and mechanical performance.

The agreement between the trends observed in this study and those reported in the literature provides indirect validation of the numerical results. While physical experimentation could further enhance validation, it is beyond the scope of the present case study, which aims to demonstrate a methodology for integrating generative design, manufacturing constraints, and numerical evaluation in gear-wheel design.

## 5. Conclusions

This paper presented a numerical case study on the application of generative design to the shape optimisation of a spur gear body, considering manufacturing constraints and material properties. A finite element-based generative design workflow was applied to evaluate weight reduction and stiffness performance under different manufacturing routes and loading strategies.

The main conclusions of the study can be summarised as follows:

- The application of generative design enabled a gear-body mass reduction of up to 37.46–45.68% compared to the reference geometry, while maintaining acceptable stiffness and deformation limits;
- Designs constrained for additive manufacturing exhibited the highest potential for weight reduction, making this approach suitable for lightweight gear applications and prototype development;
- Machining-constrained designs resulted in lower deformation values and higher structural stiffness, indicating their suitability for precision applications requiring higher load-bearing capacity;
- Casting-oriented constraints led to more conservative geometries with locally reinforced regions, which is consistent with requirements for mass-produced gear wheels subjected to higher operational loads;
- The results confirm that manufacturing route acts as an independent design variable within the generative design process, significantly influencing the resulting geometry and mechanical response.

Compared to conventional optimisation methods, generative design enables a broader exploration of shape alternatives by automatically generating multiple design variants based on defined boundary conditions and manufacturing constraints. While traditional parametric optimisation typically relies on predefined geometries and limited design iterations, generative design can discover unconventional yet efficient forms that would be difficult to conceive manually. In the context of gear wheel design, this approach provides improved material efficiency and potential weight reduction while maintaining required strength and functionality. Therefore, generative design offers a promising methodology for complex mechanical components, complementing and extending the capabilities of classical optimisation techniques.

The presented findings provide practical guidance for the selection of generative design settings in relation to different manufacturing technologies. Although the study is limited to numerical analysis, the proposed methodology and observed trends may support early-stage design decisions for gear wheels. Furthermore, considerations related to production costs, choice of manufacturing technology, and potential sustainability implications should be integrated into the decision-making process. Future work will focus on experimental validation and the extension of the approach to fatigue and dynamic performance assessment. The presented results are derived from a numerical case study and are intended to provide orientation and comparative insight into manufacturing-aware generative design of gear bodies rather than universal design rules.

## Data Availability

The original contributions presented in this study are included in the article. Further inquiries can be directed to the corresponding author.
